# Patients With Stricturing or Penetrating Crohn’s Disease Phenotypes Report High Disease Burden and Treatment Needs

**DOI:** 10.1093/ibd/izac162

**Published:** 2022-07-26

**Authors:** Yanni Fan, Ling Zhang, Negar Omidakhsh, Rhonda L Bohn, Jennifer S Thompson, Kimberly G Brodovicz, Parakkal Deepak

**Affiliations:** Boehringer Ingelheim Corporation, Ridgefield, CT, USA; Boehringer Ingelheim Corporation, Ridgefield, CT, USA; Bohn Epidemiology, Boston, MA, USA; Bohn Epidemiology, Boston, MA, USA; Boehringer Ingelheim Corporation, Ridgefield, CT, USA; Boehringer Ingelheim Corporation, Ridgefield, CT, USA; Inflammatory Bowel Diseases Center, Washington University School of Medicine in St Louis, St Louis, MO, USA

**Keywords:** biologics, Crohn’s disease complications, penetrating Crohn’s disease, stricturing Crohn’s disease

## Abstract

**Background:**

Crohn’s disease (CD) is a chronic autoimmune disease in which inflammation can progress to complications of stricturing and/or penetrating disease. Real-world data on burden of complicated CD phenotypes are limited.

**Methods:**

We analyzed cross-sectional data from the SPARC IBD (Study of a Prospective Adult Research Cohort with Inflammatory Bowel Disease) registry from 2016 to 2020. Four mutually exclusive phenotype cohorts were created: inflammatory CD (CD-I), complicated CD (stricturing CD, penetrating CD, and stricturing and penetrating CD [CD-SP]). Statistical analyses were performed using CD-I as the reference.

**Results:**

A total of 1557 patients were identified: CD-I (n = 674, 43.3%), stricturing CD (n = 457, 29.4%), penetrating CD (n = 166, 10.7%), and CD-SP (n = 260, 16.7%). Patients with complicated phenotypes reported significantly greater use of tumor necrosis factor inhibitors (84.2%-86.7% vs 66.0%; *P* < .001) and corticosteroids (75.3%-82.7% vs 68.0%; *P* < .001). Patients with CD-SP reported significantly more aphthous ulcer (15.4% vs 10.5%; *P* < .05), erythema nodosum (6.5% vs 3.6%; *P* < .05), inflammatory bowel disease-related arthropathy (25.8% vs 17.2%; *P* < .01), liquid stools (24.2% vs 9.3%; *P* < .001), nocturnal fecal incontinence (10.8% vs 2.5%; *P* < .001), and CD-related surgery (77.7% vs 12.2%; *P* < .001).

**Conclusions:**

Patients with complicated CD phenotypes reported higher rates of active CD-related luminal and extraintestinal manifestations, and underwent more surgeries, despite being more likely to have received biologics than those with CD-I. The potential for early recognition and management of CD-I to prevent progression to complicated phenotypes should be explored in longitudinal studies.

## Graphical Abstract

**Graphical Abstract:**
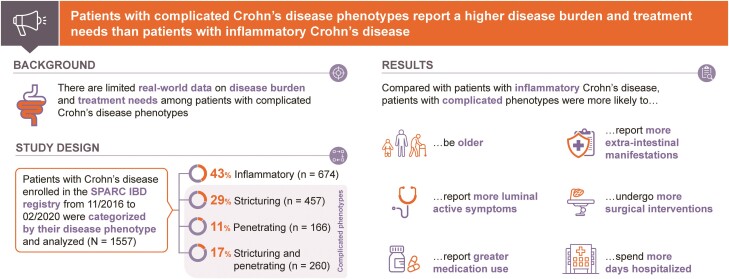

**What You Need to Know**

**Background:** There are limited real-world data describing the characteristics, treatment needs, and disease burden of patients with complicated (stricturing and/or penetrating) Crohn’s disease phenotypes.

**Findings:** Patients with complicated Crohn’s disease phenotypes have a higher disease burden, despite greater use of tumor necrosis factor inhibitors and corticosteroids, than patients with inflammatory Crohn’s disease.

**Implications for patient care:** Early recognition and optimized management of Crohn’s disease are necessary to prevent progression to complicated phenotypes and, consequently, improve health outcomes in patients with Crohn’s disease.

## Introduction

Crohn’s disease (CD) is a chronic, transmural, progressive autoimmune disease of the gastrointestinal (GI) tract with increasing global incidence and prevalence.^1-3^ The chronic inflammation that characterizes CD has a relapsing and remitting course and can progress to complications of a stricturing phenotype (fibrosis and luminal narrowing), penetrating phenotype (fistulas and/or intra-abdominal abscess), or both.^4,5^

It is estimated that half of adult patients with CD develop intestinal complications, such as strictures and fistulas, within 20 years of diagnosis, which can significantly impact their quality of life.^6,7^ In patients with a stricturing phenotype, the persistent and chronic inflammation results in fibrosis and subsequent luminal narrowing, which poses difficulty for the passage of enteric contents.^5,8^ The symptoms of strictures are cramping, abdominal pain, vomiting, and constipation; in severe cases, bowel obstruction and the need for surgical treatment can result.^1,7^ In patients with a penetrating phenotype, persistent and chronic transmural inflammation results in the development of a sinus or fistulous tract with or without abscess, which leads to symptoms ranging from abdominal pain and diarrhea to fatigue and fever, resulting in a high disease burden and the need for surgery.^1,5,8,9^ The considerable burden of this complex disease among patients also has a substantial social and economic impact.^10,11^

Currently, real-world data on disease burden and treatment needs among patients with complicated (stricturing and/or penetrating) CD phenotypes are limited. Evaluating the population of patients with CD with complicated phenotypes and understanding the disease burden are critical for the development of targeted interventions and treatments to aid in the prevention and management of the complications of CD.

The aim of this study is to describe the characteristics, comorbidities, symptoms, management (medical and surgical), and healthcare resource utilization of patients with CD with complicated phenotypes (stricturing CD [CD-S], penetrating CD [CD-P], and stricturing and penetrating CD [CD-SP]) compared with the inflammatory phenotype (inflammatory CD [CD-I]) in the SPARC IBD (Study of a Prospective Adult Research Cohort with Inflammatory Bowel Disease) registry.

## Methods

### Study Data Source

This is a cross-sectional, observational study of data collected from a multicenter, prospective, longitudinal registry that enrolled adult patients with inflammatory bowel disease (IBD) (SPARC IBD registry).^12^ The data were collected at 17 clinical sites across the United States from November 2016 to February 2020.

The SPARC IBD registry, a distinct cohort within the IBD Plexus platform, was initiated in November 2016 by the Crohn’s and Colitis Foundation. Data were collected through electronic case report forms (eCRFs), the SPARC IBD SmartForm (SSF) (completed by patients attending outpatient appointments for IBD care and those participating in a quarterly survey), patient-reported outcomes, prespecified biological samples, and routine care visits, and stored in the SPARC IBD registry. The SSF captures data from patients that include CD diagnosis, symptoms, disease assessment, severity scores (including short Crohn’s Disease Activity Index [sCDAI]), surgical history, extraintestinal manifestations, medication history, treatments, and disease phenotype. Further details of the registry and patient cohort have been published previously.^12^

In this study, we examined data collected in the eCRF and SSF at the time of enrollment to the SPARC IBD registry (defined as any time within 30 days of patient consent) and extracted on February 18, 2020.

### Study Population

Eligible patients were ≥18 years of age and had a confirmed CD diagnosis and a physician-reported CD phenotype. Patients were categorized into 1 of 4 mutually exclusive phenotype cohorts (CD-I, CD-S, CD-P, or CD-SP), defined in accordance with the Montreal classification system.^13^ Perianal disease could include perianal fistulizing disease; therefore, perianal fistulas were not necessarily classified as fistulizing CD.

To reduce the likelihood of misclassification of CD phenotypes from the registry, the phenotypes were defined based on physician-reported responses at the time of enrollment (as defined previously). Phenotypes were defined using the carry-forward model, with severity ranked as CD-SP > CD-S or CD-P > CD-I. Based on subsequent phenotype observations, a patient with CD-S or CD-P at one time point could be reclassified as CD-SP, but not as CD-I, while a patient with CD-SP at one time point retained this classification regardless of any subsequent observations. The CD-I phenotype was defined using a carry-backward method, whereby a patient classified as having CD-I at any time point was assigned to the CD-I cohort regardless of any prior classification as unknown or missing. In addition, we excluded anal strictures when defining CD-S and CD-SP, while CD-P and CD-SP were defined based on a history of fistula or abscess.

For each of the 4 CD phenotypes, the demographics and clinical characteristics, medical conditions, IBD-related conditions and symptoms, IBD medications and treatments, and healthcare resource utilization outcomes were reported from the time of enrollment (as described previously).

### Statistical Analysis

Data outcomes were reported from the time of enrollment and evaluated separately for each of the 4 phenotypes. Analyses for this study were primarily descriptive in nature, although statistical comparisons were performed on outcomes for which there were adequate data (<50% missing data). Patients with invalid data were defined as those who reported unknown or not tested for that specific outcome, and patients with missing data were defined as those who did not complete a survey form. In the descriptive analysis, the results for numeric outcomes were obtained from patients with no missing data. For binary or categorical outcomes, the denominator for percentage calculation was the total number of patients in each cohort. Missing rates were provided per variable when applicable. Outcomes were only compared between complicated phenotypes (CD-S, CD-P, and CD-SP) and the CD-I phenotype (ie, the CD-I phenotype was used as the reference). For outcomes with a binary variable, logistic regression analysis was used; for a multicategory variable, multinomial logistic regression was used; and for a numerical variable, linear regression was used to generate statistical significance of the comparisons. All statistical analyses were performed using R version 3.6.3 (R Foundation for Statistical Computing, Vienna, Austria).

### Study Reporting and Approval

The results of this study are reported in accordance with the STROBE (Strengthening the Reporting of Observational Studies in Epidemiology) guidelines for cohort studies.

This study utilized secondary registry data, with no identifiable patient information. As such, no Institutional Review Board approval was required.

## Results

### Demographics and Characteristics

**Figure 1.**
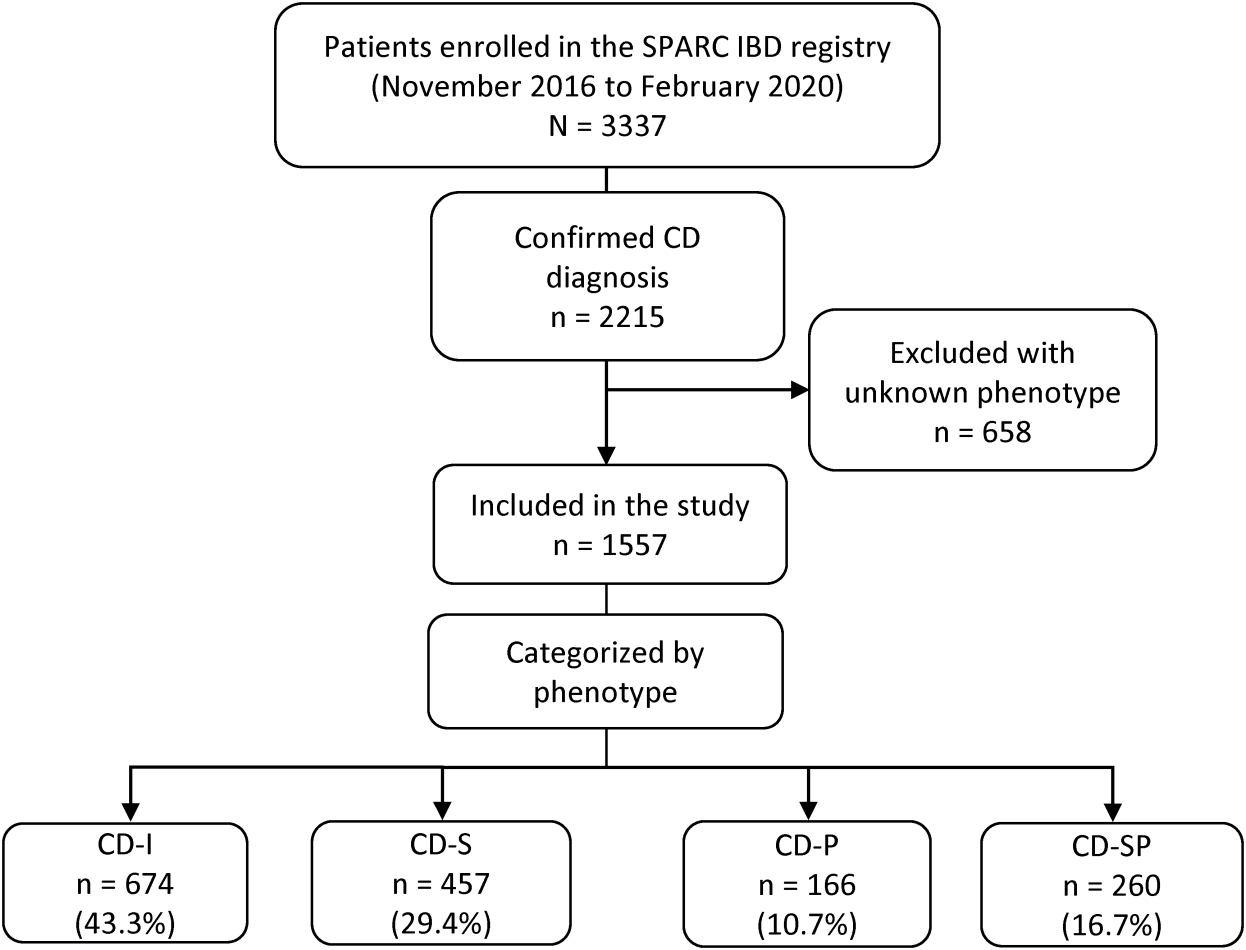
Study population. CD, Crohn’s disease; CD-I, inflammatory Crohn’s disease; CD-P, penetrating Crohn’s disease; CD-S, stricturing Crohn’s disease; CD-SP, structuring and penetrating Crohn’s disease; SPARC IBD, Study of a Prospective Adult Research Cohort with Inflammatory Bowel Disease.

In the SPARC IBD registry, at the time of data extraction, there were 3337 enrolled patients with IBD (either ulcerative colitis or CD) (**Figure 1**). There were 2215 patients with a confirmed CD diagnosis, 658 of whom had an unknown phenotype and were excluded from further analysis. The demographics and clinical characteristics of these 658 patients are shown in Supplementary Table 1. All patients with CD in the SPARC IBD registry with a known phenotype were included in the study, totaling 1557 patients across the 4 phenotypes (CD-I [n = 674, 43.3%], CD-S [n = 457, 29.4%], CD-P [n = 166, 10.7%], and CD-SP [n = 260, 16.7%]).

Overall, across the 4 CD phenotypes, patients were more likely to be female (55.6%-60.0%), except for patients with CD-P (42.8%), and White (75.9%-84.2%) (Table 1). Patients with complicated phenotypes had a longer mean CD duration (15.5-19.1 years vs 12.6 years) than patients with CD-I. Tobacco use during the 3-month period before enrollment was reported in a small percentage of patients (5.9%-8.1%) with each phenotype.

**Table 1.** Demographics and Clinical Characteristics of Patients With CD at Enrollment

Characteristic	CD-I (n = 674)	CD-S (n = 457)	CD-P (n = 166)	CD-SP (n = 260)
Age, y	38.9 ± 14.8	43.5 ± 14.5	39.8 ± 12.7	42.0 ± 13.2
Female	375 (55.6)	274 (60.0)	71 (42.8)	155 (59.6)
Race				
Black or African American	48 (7.1)	26 (5.7)	19 (11.4)	22 (8.5)
White	546 (81.0)	384 (84.0)	126 (75.9)	219 (84.2)
Other and unknown	80 (11.9)	47 (10.3)	21 (12.7)	19 (7.3)
Ethnicity				
Hispanic or Latinx	11 (1.6)	5 (1.1)	0	2 (0.8)
Not Hispanic or Latinx	663 (98.4)	452 (98.9)	166 (100.0)	258 (99.2)
BMI, kg/m^2^^a^	27.2 ± 6.1	26.8 ± 5.9	28.5 ± 6.8	27.7 ± 7.4
Hemoglobin, g/dL^b^	13.2 ± 2.0	13.0 ± 1.9	13.0 ± 1.9	14.6 ± 22.2
Albumin, g/dL^c^	4.7 ± 8.6	4.9 ± 11.0	6.3 ± 17.8	5.3 ± 13.0
CRP, mg/L^d^	25.9 ± 39.6	23.8 ± 39.2	24.0 ± 37.8	17.7 ± 33.5
Fecal calprotectin, μg/g^e^	103.8 ± 117.8	109.9 ± 117.5	61.2 ± 88.6	83.1 ± 103.4
CD duration, y^f^	12.6 ± 10.0	18.8 ± 11.4	15.5 ± 9.6	19.1 ± 11.8
Sites of CD onset				
Upper GI tract^g^	79 (11.7)	93 (20.4)^h^	32 (19.3)^i^	54 (20.8)^h^
Ileal	102 (15.1)	140 (30.6)^h^	31 (18.7)	63 (24.2)^j^
Ileocolonic	167 (24.8)	130 (28.4)	59 (35.5)^j^	104 (40.0)^h^
Colonic	97 (14.4)	15 (3.3)^h^	11 (6.6)	8 (3.1)^h^
Perianal^k^	79 (11.7)	66 (14.4)	60 (36.1)^h^	94 (36.2)^h^
sCDAI^l^	146.3 ± 90.1	156.8 ± 94.3	146.3 ± 84.1	158.1 ± 96.7
Disease severity by sCDAI^l^				
Remission	404 (59.9)	270 (59.1)	110 (66.3)	152 (58.5)
Mild	140 (20.8)	88 (19.3)	25 (15.1)	47 (18.1)
Moderate	98 (14.5)	83 (18.2)	30 (18.1)	48 (18.5)
Severe	8 (1.2)	7 (1.5)	0	5 (1.9)
Tobacco use in last 3 mo^m^	40 (5.9)	37 (8.1)	13 (7.8)	21 (8.1)
Corticosteroids ≥ 10 mg/d for ≥ 60 d^n^	28 (4.2)	13 (2.8)	7 (4.2)	7 (2.7)

Values are mean ± SD or n (%).

Abbreviations: BMI, body mass index; CD, Crohn’s disease; CD-I, inflammatory Crohn’s disease; CD-P, penetrating Crohn’s disease; CD-S, stricturing Crohn’s disease; CD-SP, stricturing and penetrating Crohn’s disease; CRP, C-reactive protein; GI, gastrointestinal; sCDAI, short Crohn’s Disease Activity Index.

^a^Data not available for 41.4%-47.6% of patients.

^b^Data not available for 35.0%-45.2% of patients.

^c^Data not available for 51.5%-61.1% of patients.

^d^Data not available for 77.1%-81.2% of patients.

^e^Data not available for 65.9%-73.5% of patients.

^f^Data not available for 5.7%-10.1% of patients.

^g^Included 0.9%-5.7% patients with only upper GI tract site of CD onset.

^h^ *P* < .001 vs CD-I.

^i^ *P *<* *.05 vs CD-I.

^j^ *P *<* *.01.

^k^Included 3.1%-11.9% patients with only perianal site of CD onset.

^l^Data not available for 0.6%-3.6% of patients.

^m^Data not available 39.6%-49.4% of patients.

^n^Data not available 10.8%-21.7% of patients.

CD onset was typically reported in the ileocolonic (24.8% CD-I; 35.5% CD-P; 40.0% CD-SP) or ileal (30.6% CD-S) locations. A significantly higher proportion of patients with complicated phenotypes than CD-I reported upper GI tract involvement at the time of disease onset (19.3%-20.8% vs 11.7% CD-I; *P* < .05 for all comparisons) (Table 1). Furthermore, a higher proportion of patients with complicated phenotypes had ileal (30.6% CD-S and 24.2% CD-SP vs 15.1% CD-I) and perianal (36.1% CD-P and 36.2% CD-SP vs 11.7%) sites of disease at onset than patients with CD-I (*P* < .01 for all comparisons). CD severity score, as measured by the sCDAI, was comparable across all phenotypes (mean score: 146.3-158.1).

### Clinical Characteristics

Patients with complicated phenotypes were more likely to report night-time awakening to move bowels (21.4%-30.8% vs 16.8% CD-I), mostly or all liquid stools (19.9%-24.2% vs 9.3% CD-I), and fecal incontinence during sleep (10.8%-11.4% vs 2.5% CD-I) compared with patients with CD-I (*P* < .05 for all comparisons) (Table 2). A numerically higher proportion of patients with complicated phenotypes reported a degree of fecal urgency as a symptom compared with patients with CD-I (57.9%-66.7% vs 53.5% CD-I). Moreover, a significantly higher proportion of patients with CD-SP reported mild (35.0% vs 31.0% CD-I) and moderate-to-moderately severe (26.7% vs 19.0% CD-I) urgency before bowel movements compared with patients with CD-I (*P* < .05 for each comparison) (Table 2). Levels of abdominal pain were broadly consistent across phenotypes, with no statistically significant differences observed.

**Table 2.** Patient-Reported Symptoms by CD Phenotype at Enrollment

Symptom	CD-I (n = 674)	CD-S (n = 457)	CD-P (n = 166)	CD-SP (n = 260)
Frequency of daily bowel movements in prior week^a^	2.3 ± 1.8	3.0 ± 2.2^b^	2.8 ± 2.0^c^	3.1 ± 2.4^b^
Frequency of current bowel movements^d^	3.4 ± 3.0	4.2 ± 3.5^b^	3.9 ± 3.3	4.4 ± 4.0^b^
General well-being in the prior week^e^				
Generally well or slightly under par	570 (84.6)	394 (86.2)	145 (87.3)	221 (85.0)
Poor, very poor, or terrible	82 (12.2)	56 (12.3)	21 (12.7)	34 (13.1)
Change in daily stool frequency in the prior week^f^				
Normal	414 (61.4)	272 (59.5)	113 (68.1)	168 (64.6)
1 or 2 stools/d > normal	129 (19.1)	87 (19.0)	30 (18.1)	44 (16.9)
3 or 4 stools/d > normal	63 (9.3)	49 (10.7)	5 (3.0)^c^	23 (8.8)^c^
5 + stools/d > normal	46 (6.8)	40 (8.8)	17 (10.2)	18 (6.9)
Consistency of stools in the prior week^g^				
Formed	206 (30.6)	106 (23.2)	43 (25.9)	49 (18.8)
Soft or semi-formed	220 (32.6)	140 (30.6)	58 (34.9)	82 (31.5)^c^
Mostly or all liquid	63 (9.3)	105 (23.0)^b^	33 (19.9)^b^	63 (24.2)^b^
Night-time awakening to move bowels in the prior month^h^	113 (16.8)	98 (21.4)^c^	46 (27.7)^c^	80 (30.8)^b^
Fecal incontinence during sleep in the prior month^i^	17 (2.5)	50 (10.9)^b^	19 (11.4)^b^	28 (10.8)^b^
Fecal incontinence while awake in the prior month^j^	50 (7.4)	62 (13.6)^b^	19 (11.4)	39 (15.0)^b^
Unintentional weight loss in the prior 6 mo	68 (10.1)	50 (10.9)	23 (13.9)	27 (10.4)
Urgency before bowel movements in the prior week^k^	n = 664	n = 446	n = 159	n = 240
Mild	206 (31.0)	139 (31.2)	44 (27.7)	84 (35.0)^c^
Moderate-to-moderately severe	126 (19.0)	120 (26.9)^b^	38 (23.9)	64 (26.7)^b^
Severe	23 (3.5)	25 (5.6)^c^	10 (6.3)	12 (5.0)
No urgency	289 (43.5)	153 (34.3)	66 (41.5)	74 (30.8)
Abdominal pain by sCDAI^l^				
Mild	208 (30.9)	126 (27.6)	39 (23.5)	78 (30.0)
Moderate	112 (16.6)	67 (14.7)	22 (13.3)	35 (13.5)
Severe	22 (3.3)	20 (4.4)	8 (4.8)	9 (3.5)
None	311 (46.1)	237 (51.9)	97 (58.4)	133 (51.2)

Values are mean ± SD or n (%).

^a^Data missing for 3.0%-5.5% of patients.

^b^ *P* < .001 vs CD-I.

^c^ *P* < .05 vs CD-I.

^d^Data not available for 0.6%-4.2% of patients.

^e^Data not available for 0.0%-3.3% of patients.

^f^Data not available for 0.6%-3.3% of patients.

^g^Data not available for 19.3%-27.4% of patients.

^h^Data not available for 19.3%-27.6% of patients.

^i^Data not available for 19.3%-27.6% of patients.

^j^Data not available for 19.9%-27.6% of patients.

^k^Data not available for 0.6%-3.0% of patients.

^l^Data not available for 0.0%-3.1% of patients.

Abbreviations: CD, Crohn’s disease; CD-I, inflammatory Crohn’s disease; CD-P, penetrating Crohn’s disease; CD-S, stricturing Crohn’s disease; CD-SP, stricturing and penetrating Crohn’s disease; sCDAI, short Crohn’s Disease Activity Index.

Of all the medical conditions relating to disease reported at enrollment, patients with all CD phenotypes were most likely to report IBD-related arthropathy, with the greatest proportion reported in patients with CD-SP (25.8%) (Table 3). Patients with CD-SP had a significantly higher prevalence of IBD-related arthropathy (25.8% vs 17.2% CD-I), aphthous ulcer (15.4% vs 10.5% CD-I), erythema nodosum (6.5% vs 3.6% CD-I), pyoderma gangrenosum (4.2% vs 1.0% CD-I), thrombotic complications (5.0% vs 2.1% CD-I), and personal history of cervical dysplasia (3.1% vs < 1% CD-I) compared with patients with CD-I (*P* < .05 for each comparison).

**Table 3.** History of Medical Conditions by CD Phenotype Reported at Enrollment

Medical Condition	CD-I (n = 674)	CD-S (n = 457)	CD-P (n = 166)	CD-SP (n = 260)
IBD-related arthropathy	116 (17.2)	91 (19.9)	31 (18.7)	67 (25.8)^a^
Aphthous ulcer	71 (10.5)	37 (8.1)	19 (11.4)	40 (15.4)^b^
Erythema nodosum	24 (3.6)	12 (2.6)	5 (3.0)	17 (6.5)^b^
Thrombotic complications	14 (2.1)	9 (2.0)	3 (1.8)	13 (5.0)^b^
Iritis/uveitis	34 (5.0)	13 (2.8)	6 (3.6)	11 (4.2)
Pyoderma gangrenosum	7 (1.0)	7 (1.5)	6 (3.6)^b^	11 (4.2)^a^
History of cervical dysplasia	6 (0.9)	8 (1.8)	2 (1.2)	8 (3.1)^b^
Any intestinal dysplasia	13 (1.9)	5 (1.1)	3 (1.8)	5 (1.9)
Primary sclerosing cholangitis	14 (2.1)	6 (1.3)	2 (1.2)	3 (1.2)
Malignant neoplasm of colon, unspecified	2 (0.3)	3 (0.7)	1 (0.6)	0

Values are n (%). Variables were created based on the reported history of respective phenotypes, if there was a record of the disease any time before the index date until the index date +30 days.

Abbreviations: CD, Crohn’s disease; CD-I, inflammatory Crohn’s disease; CD-P, penetrating Crohn’s disease; CD-S, stricturing Crohn’s disease; CD-SP, stricturing and penetrating Crohn’s disease; IBD, inflammatory bowel disease.

^a^ *P* < .01 vs CD-I.

^b^ *P* < .05 vs CD-I.

### Treatment

**Figure 2.**
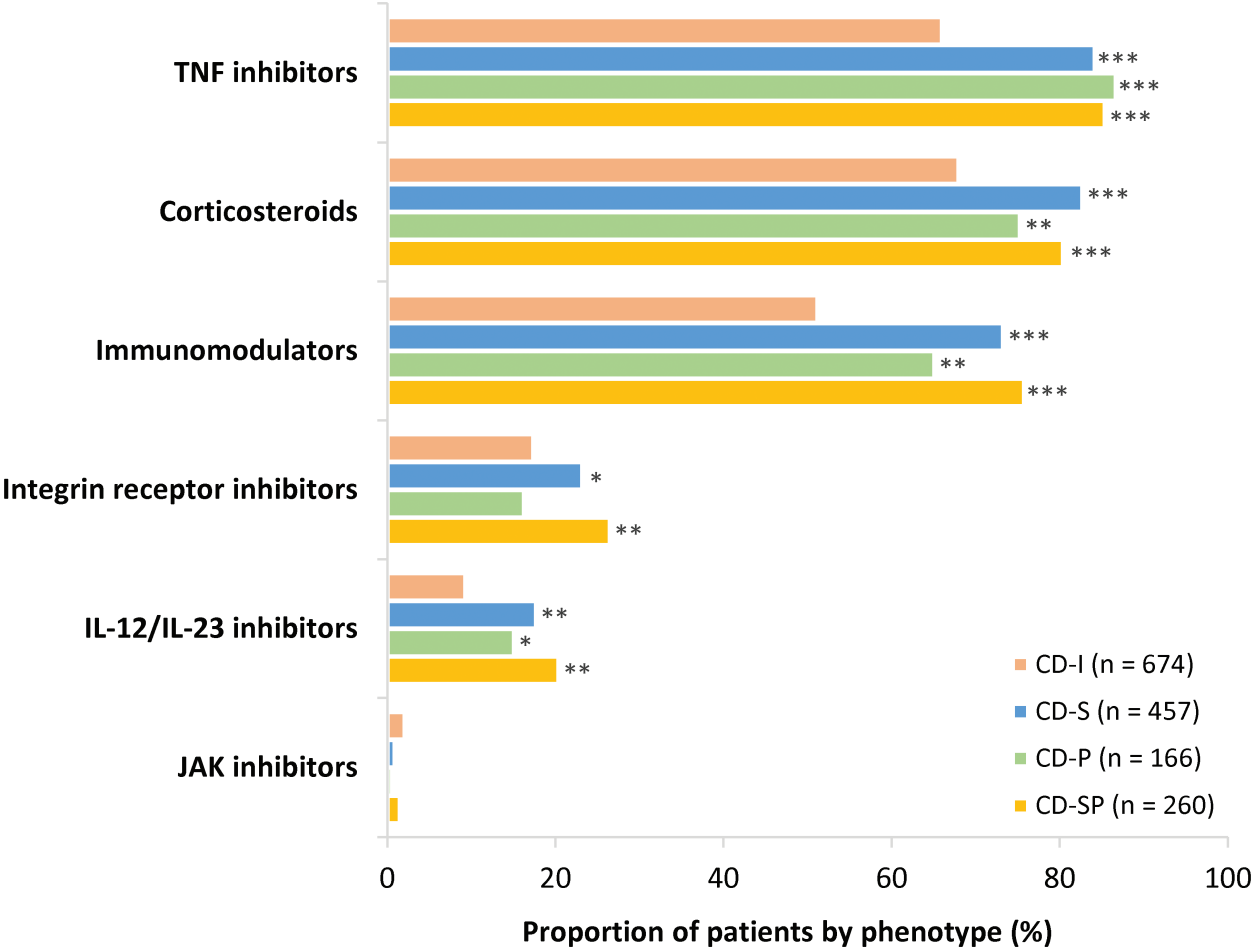
History of medication use at class level by Crohn’s disease phenotype at enrollment. Medications were those that patients either were taking at the time of enrollment or had taken at any time previously. *P < .05 vs CD-I; **P < .01 vs CD-I; ***P < .001 vs CD-I. Medication classes are not mutually exclusive. CD-I, inflammatory Crohn’s disease; CD-P, penetrating Crohn’s disease; CD-S, stricturing Crohn’s disease; CD-SP, stricturing and penetrating Crohn’s disease; IL, interleukin; JAK, Janus kinase; TNF, tumor necrosis factor.

Patients’ use of treatment by drug class is summarized in Figure 2. The IBD medications included within each medication class are listed in Supplementary Table 2, and patients’ use of tumor necrosis factor (TNF) inhibitors alone or in combination with other treatments is shown in Supplementary Table 3. Individuals with complicated phenotypes reported higher use of all IBD medications at the class level compared with patients with CD-I. The only exception was treatment with the Janus kinase inhibitor class; however, reported use of this class was by <5% of patients across all phenotypes. Patients with complicated phenotypes were significantly more likely to report using TNF inhibitors (84.2%-86.7% vs 66.0% CD-I) and corticosteroids (75.3%-82.7% vs 68.0% CD-I) compared with patients with CD-I (*P* < .001 for all comparisons). The percentage of patients who had taken corticosteroids at a dose of at least 10 mg/d for 60 days or more at the time of enrollment was 4.2% in the CD-I and CD-P groups, and 2.7% and 2.8% in CD-SP and CD-S groups, respectively.

Medication use at the drug level by phenotype is presented in Supplementary Figure 1, and patients’ use of vedolizumab and ustekinumab alone or in combination with other treatments is shown in Supplementary Table 3. A significantly higher proportion of patients with complicated phenotypes reported using corticosteroids (74.1%-81.0% vs 65.9% CD-I), thiopurines (55.4%-66.2% vs 42.1% CD-I), infliximab (57.1%-66.2% vs 37.8% CD-I), and adalimumab (51.8%-60.2% vs 43.2% CD-I) compared with patients with CD-I (*P* < .05 for all comparisons).

**Figure 3.**
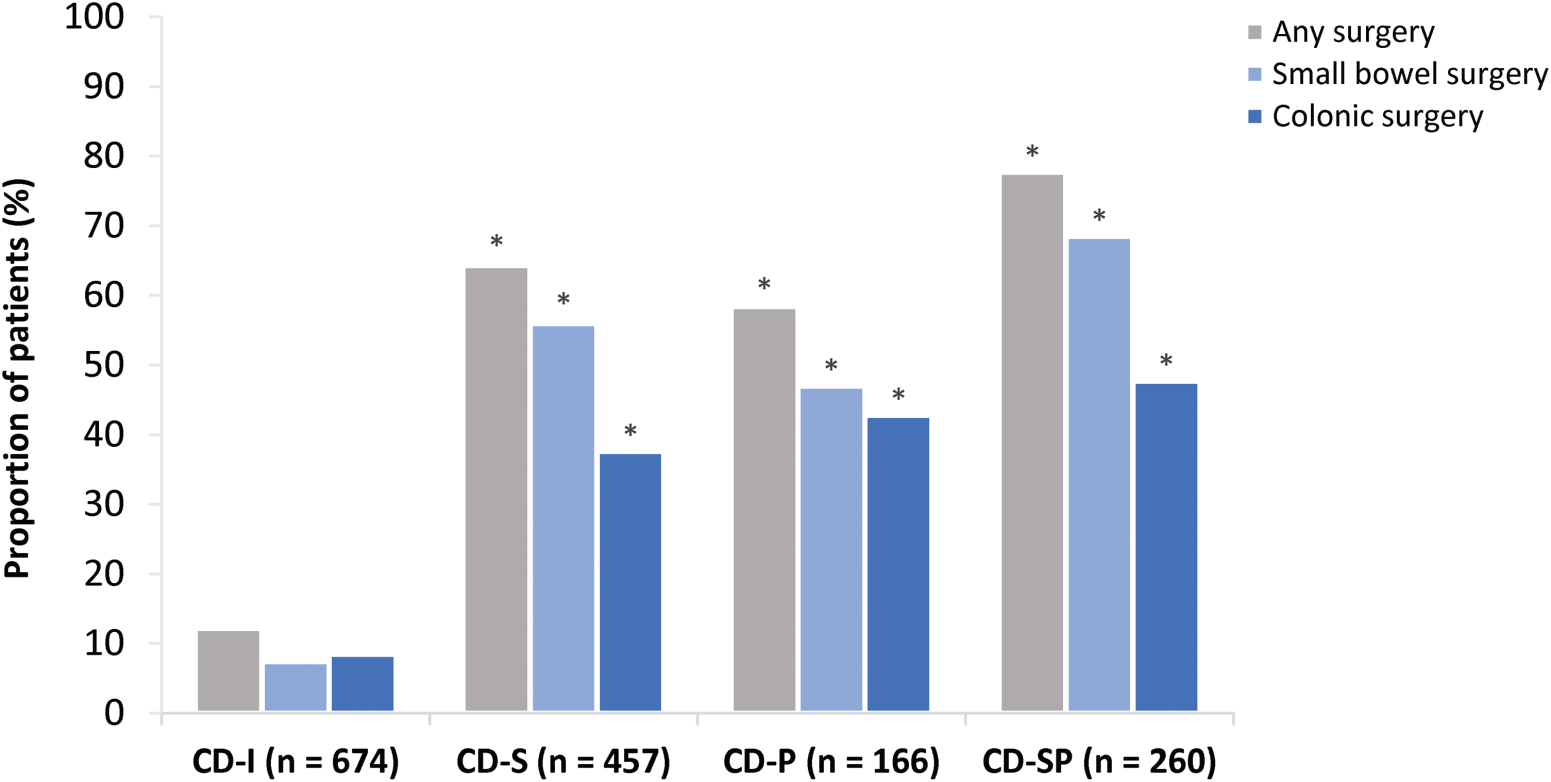
Surgeries by Crohn’s disease (CD) phenotype at enrollment. Only luminal CD surgery was recorded; perianal disease-specific procedures were not recorded. *P < .001 vs inflammatory CD (CD-I) for all comparisons. Missing values are not shown; therefore, values may not add up to 100%. Surgery categories are not mutually exclusive. Any surgery indicates as small bowel resection, colonic or complete colectomy; small bowel surgery indicates small bowel resection only; colonic surgery indicates colonic and complete colectomy (whole colon removed). CD-P, penetrating Crohn’s disease; CD-S, stricturing Crohn’s disease; CD-SP, stricturing and penetrating Crohn’s disease.

Patients with complicated phenotypes received significantly more surgical interventions than patients with CD-I; these interventions included any CD-related surgery (58.4%-77.7% vs 12.2%), including small bowel (47.0%-68.5% vs 7.4%) and colonic (37.6%-47.7% vs 8.5%) surgery (*P* < .001 for all comparisons) (Figure 3).

### Healthcare Resource Utilization and Productivity Loss

Numerically higher percentages of patients with complicated phenotypes vs CD-I required ≥1 day of hospitalization in the past 6 months (3.1%-5.0% vs 1.5% CD-I) (Supplementary Table S4). However, no such difference was evident in the percentages of patients missing ≥1 work or school day due to IBD-related illness in the prior 6 months (4.8%-9.0% vs 6.5% vs CD-I).

The mean number of missed days of work or school due to IBD during the prior 6 months was numerically higher in patients with CD-SP or CD-P (~26 days) than in those with CD-I or CD-S (~5-7 days). Patients with CD-SP reported a numerically higher mean number of days hospitalized due to IBD during the prior 6 months than in patients with the other phenotypes (11.1 days, compared with 3.8-7.3 days among patients with CD-I, CD-S or CD-P).

## Discussion

Although approximately half of patients with CD are estimated to progress from inflammatory to complicated phenotypes within 20 years of diagnosis,^6^ real-world evidence from patients with complicated CD phenotypes is limited. Thus, there is a gap in our understanding of the disease characteristics, outcomes, and burden of patients with CD.

In this study, we examined a range of characteristics of patients with CD within 4 distinct, physician-reported phenotype cohorts (CD-I, CD-S, CD-P, and CD-SP) enrolled in the U.S.-based, multicenter SPARC IBD registry. Unique aspects of the study design were the prospective collection of data on patient-reported signs and symptoms of luminal and extraintestinal manifestations of CD, and how these symptoms related to the physician-reported CD phenotypes. Patients with complicated CD were generally more likely to report active and severe CD-related symptoms than patients with CD-I. These include a greater frequency of mild and moderate-to-moderately severe urgency before bowel movements and an increased occurrence of mostly or all liquid stools in the prior week, and increased night-time awakening to move bowels and increased fecal incontinence (during sleep and while awake) in the prior month. Critically, these patient-reported symptoms, which, in our study, were more frequent in complicated than inflammatory CD, are not typically captured by other measures of disease activity, yet have a considerable detrimental impact on patient quality of life and daily functioning.^14,15^

Regarding extraintestinal manifestations of CD, in our study, the most common concomitant medical condition at enrollment reported among all phenotype cohorts was IBD-related arthropathy. Patients with complicated CD, specifically those with CD-SP, reported more IBD-related arthropathy, aphthous ulcer, erythema nodosum, pyoderma gangrenosum, and thrombotic complications than patients with CD-I. A significantly increased history of cervical dysplasia was also found in patients with CD-SP vs CD-I. The cause of this is unclear, but a potential association between reported immunomodulators and prior cervical dysplasia cannot be definitively excluded^16,17^; significantly more patients with CD-SP vs CD-I reported the use of immunomodulators.

Notably, the higher rates of patient-reported symptoms and extraintestinal manifestations in the complicated CD phenotype cohorts were evident despite these patients receiving TNF inhibitors, corticosteroids, and immunomodulators significantly more often than patients with CD-I. The reported use of the Janus kinase inhibitor class was by <5% of patients across all phenotypes, likely reflecting the 2018 approval of tofacitinib for use in ulcerative colitis.^18,19^ Phenotype-specific differences between cohorts aside, the overall pattern of CD-related disease management with medication and surgery was consistent with the published literature.^3,8,20^ The greatest proportion of patients needing any surgery was patients with CD-SP. Similarly, most patients with CD-S or CD-P required at least 1 surgical resection, of which small bowel resection was the most common; conversely, most patients with CD-I had not had surgery. These data are in line with a population-based systematic review of patients with CD in North America, which reported that up to 57% of patients required at least 1 surgical resection.^3,20^ Surgical resections, especially those in the small bowel, increase the risk of short bowel syndrome and impaired gut functionality, thereby collectively impacting patient quality of life.^5,7,21^

A limitation of this study is that the use of medical records, SSFs, and eCRFs can potentially lead to missing data, under-reporting of outcomes, and inaccuracies in the data recorded. A few of the variables analyzed in our study could have up to 50% data not available (not planned for collection and only extracted from EMR), potentially confounding the results. The accuracy of the analyses depends on data being correctly documented in medical charts and recorded or coded in eCRFs and SSFs across sites. Similarly, limitations can arise from the potential misclassification of CD phenotypes from the registry, and the lack of endoscopic or imaging data in our study may be relevant in this regard. However, we used an algorithm to minimize the possibility of misclassification, and inaccuracies were likely limited using a common SSF and eCRF across sites along with a structured training process for coordinators, involving a manual created by the SPARC IBD SSF working group to improve standardization of phenotyping of participants using specific criteria. In a data quality assessment to validate the accuracy of key data elements in the SSF, agreement in CD behavior ranged from 70% to 100%, with an overall agreement in CD behavior of 83%.^12^

Finally, the SPARC IBD data are not representative of all patients in the IBD Plexus platform; rather, the data represent the cohort of patients who completed the SPARC IBD questionnaires. The data may also not be representative of patients with CD outside the IBD Plexus platform. For example, upper GI CD is usually uncommon but was present in 16.6% of patients included in our study. Also, most patients in this study were White (not Hispanic or Latinx), raising uncertainty regarding applicability of the findings to patients of other race or ethnicity. However, the noninterventional setting and broad inclusion criteria in the study ensured some similarities with real-world patients. In our study, across all CD phenotypes, except for CD-P, more than 50% of the patient population were female, which is consistent with a previous systematic review on the epidemiology of CD.^20^ Across all CD phenotypes, the site of disease onset was most commonly in the ileal or ileocolonic region, also consistent with prior studies.^3,6^ Patient sCDAI severity scores were comparable across all phenotypes, and most patients were in remission. However, the reported data for remission rates, taken at a single time point, should be interpreted with caution, as most patients with CD have a chronic intermittent disease course.^20^ Overall, patients with complicated CD phenotypes in this study were more likely to be older and have a longer disease duration than patients with an inflammatory phenotype (ie, patients with CD-I tended to have a shorter disease duration), suggesting an earlier stage in the natural history of the disease.

## Conclusions

In summary, patients with complicated CD phenotypes reported higher rates of active CD-related luminal and extraintestinal manifestations, and underwent more surgeries, despite being more likely to have received biologics than patients with CD-I. Our results highlight the high disease burden in patients with complicated phenotypes. Future studies with a larger sample size and longitudinal data are needed to assess the impact of early recognition and management of CD-I with tight control toward treatment targets, to potentially prevent progression to complications of stricturing or penetrating disease and related impact on patient-reported symptoms, work productivity, and quality of life.

## Supplementary Material

izac162_suppl_Supplementary_Material

## Data Availability

The results published here are based on data from the SPARC IBD registry obtained from the IBD Plexus platform of the Crohn’s and Colitis Foundation. People interested in accessing SPARC IBD data for research should contact the Crohn’s and Colitis Foundation. Please visit https://www.crohnscolitisfoundation.org/ for more information.
