# Supplementary material for: Patients With Stricturing or Penetrating Crohn’s Disease Phenotypes Report High Disease Burden and Treatment Needs
Source: Inflamm Bowel Dis. 2022 Jul 26;29(6):914–22. doi: 10.1093/ibd/izac162 (PMC10233399; doi:10.1093/ibd/izac162)
Supplement: izac162_suppl_Supplementary_Material [file izac162_suppl_supplementary_material.docx]

**Supplementary Materials**

**Table S1.** Demographics and Clinical Characteristics of Patients with Crohn’s Disease of Unknown Phenotype at Enrollment

| Characteristic | Patients with unknown phenotype  (N = 658) |
| --- | --- |
| Age (years), mean (SD) | 42.5 (14.3) |
| Female, n (%) | 393 (59.7) |
| Race, n (%) |  |
| Black or African American | 42 (6.4) |
| White | 405 (61.6) |
| Other and unknown | 211 (32.1) |
| Ethnicity, n (%) |  |
| Hispanic or Latinx | 5 (0.8) |
| Not Hispanic or Latinx | 653 (99.2) |
| BMI (kg/m^2^), mean (SD)^a^ | 27.4 (6.3) |
| Hemoglobin (g/dL), mean (SD)^b^ | 13.1 (1.9) |
| Albumin (g/dL), mean (SD)^c^ | 4.1 (0.5) |
| CRP (mg/L), mean (SD)^d^ | 29.2 (40.2) |
| Fecal calprotectin (μg/g), mean (SD)^e^ | 106.0 (120.0) |
| CD duration (years), mean (SD)^f^ | 16.7 (11.9) |
| Sites of CD onset, n (%) |  |
| Upper GI tract | 5 (0.8) |
| Ileal | 16 (2.4) |
| Ileocolonic | 15 (2.3) |
| Colonic | 3 (0.5) |
| Perianal | 11 (1.7) |
| sCDAI, mean (SD)^g^ | 150.7 (93.7) |
| Disease severity by sCDAI, n (%)^g^ |  |
| Remission | 361 (54.9) |
| Mild | 121 (18.4) |
| Moderate | 105 (16.0) |
| Severe | 6 (0.9) |
| Tobacco use in last 3 months, n (%)^h^ | 12 (1.8) |
| Corticosteroids ≥10 mg/day for ≥60 days, n (%)^i^ | 10 (1.5) |

Data missing for ^a^285, ^b^272, ^c^379, ^d^455, ^e^471, ^f^368, ^g^65, ^h^565, ^i^555 patients.
BMI, body mass index; CD, Crohn’s disease; CRP, C-reactive protein; GI, gastrointestinal; sCDAI, short Crohn’s Disease Activity Index; SD, standard deviation.

**Table S2.** Inflammatory Bowel Disease Medications

| Category | Medication(s) |
| --- | --- |
| Corticosteroids | Budesonide, steroid suppository, steroid enema |
| IL-12/IL-23 inhibitors | Ustekinumab |
| Immunomodulators | Methotrexate, thiopurine, azathioprine |
| Integrin receptor inhibitors | Vedolizumab, natalizumab |
| JAK inhibitors | Tofacitinib |
| TNF inhibitors | Infliximab, adalimumab, certolizumab pegol, golimumab |

IL, interleukin; JAK, Janus kinase; TNF, tumor necrosis factor.

**Table S3.** Medication Use by Crohn’s Disease Phenotype: Biologic Treatments Administered Alone or in Combination with Immunomodulators

| Treatment | CD-I  (n = 674) | CD-S  (n = 457) | CD-P  (n = 166) | CD-SP  (n = 260) | Total  (N=1557) | *P*-value |
| --- | --- | --- | --- | --- | --- | --- |
| TNF inhibitors, n (%) |  |  |  |  |  | 0.114 |
| Monotherapy | 254 (37.7) | 187 (40.9) | 69 (41.6) | 102 (39.2) | 612 (39.3) |  |
| Combined with azathioprine | 55 (8.2) | 42 (9.2) | 21 (12.7) | 23 (8.8) | 141 (9.1) |  |
| Combined with methotrexate | 25 (3.7) | 13 (2.8) | 6 (3.6) | 9 (3.5) | 53 (3.4) |  |
| Vedolizumab, n (%) |  |  |  |  |  | 0.590 |
| Monotherapy | 185 (27.4) | 146 (31.9) | 54 (32.5) | 78 (30.0) | 463 (29.7) |  |
| Combined with azathioprine | 8 (1.2) | 8 (1.8) | 1 (0.6) | 2 (0.8) | 19 (1.2) |  |
| Combined with methotrexate | 3 (0.4) | 4 (0.9) | 1 (0.6) | 2 (0.8) | 10 (0.6) |  |
| Ustekinumab, n (%) |  |  |  |  |  | 0.010 |
| Monotherapy | 189 (28.0) | 154 (33.7) | 52 (31.3) | 96 (36.9) | 491 (31.5) |  |
| Combined with azathioprine | 6 (0.9) | 11 (2.4) | 4 (2.4) | 3 (1.2) | 24 (1.5) |  |
| Combined with methotrexate | 1 (0.1) | 7 (1.5) | 3 (1.8) | 5 (1.9) | 16 (1.0) |  |

CD-I, inflammatory Crohn’s disease; CD-P, Crohn’s disease with penetrating phenotype; CD-S, Crohn’s disease with stricturing phenotype; CD-SP, Crohn’s disease with stricturing and penetrating phenotype; TNF, tumor necrosis factor.

**Table S4.** Healthcare Resource Utilization and Productivity Loss at Enrollment

|  | CD-I  (n = 674) | CD-S  (n = 457) | CD-P  (n = 166) | CD-SP  (n = 260) |
| --- | --- | --- | --- | --- |
| Missing ≥1 day of work/school due to IBD in the past 6 months, n (%)^a^ | 44 (6.5) | 22 (4.8) | 15 (9.0) | 14 (5.4) |
| *[Among patients* *with ≥1 missed day]* Number of missed days of work/school due to IBD during the prior 6 months, mean (SD) | 5.8 (5.3) | 7.6 (7.0) | 25.6 (52.4) | 26.4 (36.3) |
| Requiring hospitalization (≥1 day) in the past 6 months, n (%)^a^ | 10 (1.5) | 14 (3.1) | 6 (3.6) | 13 (5.0) |
| *[Among patients requiring ≥1 day of hospitalization]* Number of days hospitalized due to IBD during the prior 6 months, mean (SD) | 4.7 (3.7) | 7.3 (6.1) | 3.8 (1.6) | 11.1 (11.0) |

^a^Data available for all patients with each phenotype.

CD-I, inflammatory Crohn’s disease; CD-P, Crohn’s disease with penetrating phenotype; CD-S, Crohn’s disease with stricturing phenotype; CD-SP, Crohn’s disease with stricturing and penetrating phenotype; IBD, inflammatory bowel disease; SD, standard deviation.

**Figure S1.** History of Medication use at Drug Level by Crohn’s Disease Phenotype at Enrollment^a^

^a^Medications that patients were either taking at the time of enrollment or had taken at any time previously.

**P* < .05 vs CD-I; ***P* < .01 vs CD-I; ****P* < .001 vs CD-I. Medication classes are not mutually exclusive.
CD-I, inflammatory Crohn’s disease; CD-P, Crohn’s disease with penetrating phenotype; CD-S, Crohn’s disease with stricturing phenotype; CD-SP, Crohn’s disease with stricturing and penetrating phenotype.
